# 2-[(1*H*-Imidazol-2-yl)disulfan­yl]-1*H*-imidazole

**DOI:** 10.1107/S1600536811036014

**Published:** 2011-09-14

**Authors:** Mona Feizbakhsh Bazargani, Laleh Talavat, Soheila Naderi, Hamid Reza Khavasi

**Affiliations:** a Department of Chemistry, Shahid Beheshti University, G. C., Evin, Tehran 1983963113, Iran

## Abstract

In the title molecule, C_6_H_6_N_4_S_2_, a twofold rotation axis passes through the mid-point of the S—S bond. The C—S—S—C torsion angle is 83.62 (17)°. π–π stacking between imidazole rings of adjacent mol­ecules is observed in the crystal structure, the centroid–centroid distance being 3.447 (2) Å. Inter­molecular N—H⋯S hydrogen bonding results in the formation of a linear chain in the *c*-axis direction.

## Related literature

For related imidazole disulfide compounds, see: Robina *et al.* (1990 ▶); Figueroa *et al.* (2007 ▶); Chernovyants *et al.* (2008 ▶).
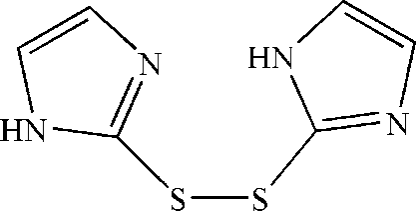


## Experimental

### 

#### Crystal data

C_6_H_6_N_4_S_2_
*M* *_r_* = 198.29
Monoclinic, 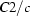

*a* = 14.083 (3) Å
*b* = 6.3928 (13) Å
*c* = 9.922 (2) Å
β = 122.29 (3)°
*V* = 755.1 (4) Å^3^
*Z* = 4
Mo *K*α radiation
μ = 0.64 mm^−1^
*T* = 298 K
0.45 × 0.25 × 0.15 mm

#### Data collection

STOE IPDS II diffractometer
Absorption correction: multi-scan (*X-RED* and *X-SHAPE*; Stoe & Cie, 2005 ▶) *T*_min_ = 0.823, *T*_max_ = 0.906
4116 measured reflections
1007 independent reflections
948 reflections with *I* > 2σ(*I*)
*R*_int_ = 0.112

#### Refinement

*R*[*F* ^2^ > 2σ(*F* ^2^)] = 0.055
*wR*(*F* ^2^) = 0.185
*S* = 1.18
1007 reflections
56 parameters
H-atom parameters constrained
Δρ_max_ = 0.82 e Å^−3^
Δρ_min_ = −0.56 e Å^−3^

### 

Data collection: *X-AREA* (Stoe & Cie, 2005 ▶); cell refinement: *X-AREA*; data reduction: *X-AREA*; program(s) used to solve structure: *SHELXS97* (Sheldrick, 2008 ▶); program(s) used to refine structure: *SHELXL97* (Sheldrick, 2008 ▶); molecular graphics: *ORTEP-3 for Windows* (Farrugia, 1997 ▶); software used to prepare material for publication: *WinGX* (Farrugia, 1999 ▶).

## Supplementary Material

Crystal structure: contains datablock(s) global, I. DOI: 10.1107/S1600536811036014/xu5318sup1.cif

Structure factors: contains datablock(s) I. DOI: 10.1107/S1600536811036014/xu5318Isup2.hkl

Supplementary material file. DOI: 10.1107/S1600536811036014/xu5318Isup3.cml

Additional supplementary materials:  crystallographic information; 3D view; checkCIF report

## Figures and Tables

**Table 1:** Hydrogen-bond geometry (Å, °)

*D*—H⋯*A*	*D*—H	H⋯*A*	*D*⋯*A*	*D*—H⋯*A*
N2—H2*A*⋯S1^i^	0.86	2.44	3.227 (3)	153

Symmetry code: (i) 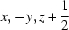.

**Fig. 1.**
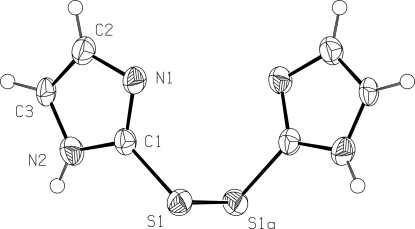
The molecular structure with the atom-numbering scheme. Displacement ellipsoids are drawn at 30% probability level.

**Fig. 2.**
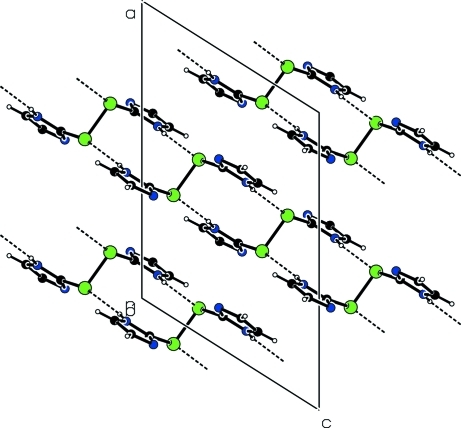
A packing diagram of (I). Hydrogen bonds are shown as dashed lines.

## supplementary crystallographic information

### Comment

There have been little attention to crystal structure determination of imidazole disulfides. The crystal structure of 2,2-Dithio-bis(1-*p*-tolyl-1*H*-imidazole-4-carboxaldehyde) (Robina *et al.*, 1990), bis(1-Phenylimidazol-2-yl)disulfide (Figueroa *et al.*, 2007), bis(1 - t-Butylimidazol-2-yl)disulfide (Figueroa *et al.*, 2007) and 2,2-Dithiobis(1-methylimidazol-3-ium-2-yl) bis(tri-iodide) di-iodine (Chernovyants *et al.*, 2008) have reported previously. Here we report the crystal structure of 2-(2-(1*H*-imidazol-2-yl)disulfanyl)-1*H*-imidazole. The asymmetric unit of the title compound, (I), contains one half-molecule and a twofold rotation axis passes through the middle of S—S bond (Fig. 1). The S—S bond distance is 2.0713 (14) Å. In this compound the imidazole rings are of course planar and the angle between these rings is 21.83 (19) °. The torsion angle of C1—S1—S1a—C1a (a: -*x*,*y*,-*z* + 1/2) is 83.62 (17) °. The ineramolecular N—H···S hydrogen bonds (Table 1) result in the formation of a linear chain in c-direction. Further pi-pi interaction between imidazole rings of adjacent chains in a-direction (cg···cg distance of 3.4466 (19) Å, *sym* code; -*x*, 1 - *y*, 1 - *z*) results in the formation of a supramolecular structure.

### Experimental

The title compound has been synthsized during the stirring of 1*H*-imidazole-2-thiol with thallium(I) acetate in 2:1 molar ration in methanol. The suitable crystals for X-ray analysis were obtained by slow evaporation from methanol solution after one week (yield; 75.5%).

### Refinement

H atoms were positioned geometrically with C—H = 0.93 and N—H = 0.86 Å, and constrained to ride on their parent atoms with *U*_iso_(H) = 1.2*U*_eq_(C,N).

### Crystal data

**Table:**

C_6_H_6_N_4_S_2_	*F*(000) = 408
*M**_r_* = 198.29	*D*_x_ = 1.744 Mg m^−^^3^
Monoclinic, *C*2/*c*	Mo *K*α radiation, λ = 0.71073 Å
Hall symbol: -C 2yc	Cell parameters from 1007 reflections
*a* = 14.083 (3) Å	θ = 3.4–23°
*b* = 6.3928 (13) Å	µ = 0.64 mm^−^^1^
*c* = 9.922 (2) Å	*T* = 298 K
β = 122.29 (3)°	Prism, colorless
*V* = 755.1 (4) Å^3^	0.45 × 0.25 × 0.15 mm
*Z* = 4	

### Data collection

**Table:**

STOE IPDS II diffractometer	1007 independent reflections
graphite	948 reflections with *I* > 2σ(*I*)
Detector resolution: 0.15 pixels mm^-1^	*R*_int_ = 0.112
rotation method scans	θ_max_ = 29.2°, θ_min_ = 3.4°
Absorption correction: multi-scan (*X-RED* and *X-SHAPE*; Stoe & Cie, 2005)	*h* = −19→19
*T*_min_ = 0.823, *T*_max_ = 0.906	*k* = −8→6
4116 measured reflections	*l* = −13→13

### Refinement

**Table:**

Refinement on *F*^2^	Secondary atom site location: difference Fourier map
Least-squares matrix: full	Hydrogen site location: inferred from neighbouring sites
*R*[*F*^2^ > 2σ(*F*^2^)] = 0.055	H-atom parameters constrained
*wR*(*F*^2^) = 0.185	*w* = 1/[σ^2^(*F*_o_^2^) + (0.1134*P*)^2^ + 0.6898*P*] where *P* = (*F*_o_^2^ + 2*F*_c_^2^)/3
*S* = 1.18	(Δ/σ)_max_ < 0.001
1007 reflections	Δρ_max_ = 0.82 e Å^−^^3^
56 parameters	Δρ_min_ = −0.56 e Å^−^^3^
0 restraints	Extinction correction: *SHELXL*
Primary atom site location: structure-invariant direct methods	Extinction coefficient: 0.11 (3)

### Special details

**Table:**

Geometry. All e.s.d.'s (except the e.s.d. in the dihedral angle between two l.s. planes) are estimated using the full covariance matrix. The cell e.s.d.'s are taken into account individually in the estimation of e.s.d.'s in distances, angles and torsion angles; correlations between e.s.d.'s in cell parameters are only used when they are defined by crystal symmetry. An approximate (isotropic) treatment of cell e.s.d.'s is used for estimating e.s.d.'s involving l.s. planes.
Refinement. Refinement of *F*^2^ against ALL reflections. The weighted *R*-factor *wR* and goodness of fit *S* are based on *F*^2^, conventional *R*-factors *R* are based on *F*, with *F* set to zero for negative *F*^2^. The threshold expression of *F*^2^ > σ(*F*^2^) is used only for calculating *R*-factors(gt) *etc*. and is not relevant to the choice of reflections for refinement. *R*-factors based on *F*^2^ are statistically about twice as large as those based on *F*, and *R*- factors based on ALL data will be even larger.

### Fractional atomic coordinates and isotropic or equivalent isotropic displacement parameters (Å^2^)

**Table:**

	*x*	*y*	*z*	*U*_iso_*/*U*_eq_	
S1	0.08686 (5)	0.07449 (9)	0.32168 (6)	0.0337 (4)	
N1	0.13136 (17)	0.4761 (4)	0.4347 (2)	0.0319 (5)	
N2	0.11134 (18)	0.2432 (4)	0.5892 (2)	0.0350 (6)	
H2A	0.1006	0.129	0.625	0.042*	
C1	0.11109 (17)	0.2743 (4)	0.4570 (2)	0.0281 (5)	
C2	0.1440 (2)	0.5734 (4)	0.5611 (3)	0.0335 (6)	
H2	0.1585	0.7155	0.5821	0.04*	
C3	0.13289 (18)	0.4382 (4)	0.6536 (2)	0.0280 (5)	
H3	0.139	0.4723	0.7491	0.034*	

### Atomic displacement parameters (Å^2^)

**Table:**

	*U*^11^	*U*^22^	*U*^33^	*U*^12^	*U*^13^	*U*^23^
S1	0.0385 (5)	0.0326 (5)	0.0294 (5)	0.00531 (19)	0.0178 (4)	−0.00166 (17)
N1	0.0407 (10)	0.0348 (11)	0.0256 (9)	−0.0026 (8)	0.0213 (7)	−0.0020 (7)
N2	0.0451 (11)	0.0375 (11)	0.0271 (10)	0.0024 (8)	0.0226 (8)	0.0042 (7)
C1	0.0300 (10)	0.0334 (11)	0.0222 (10)	0.0028 (8)	0.0147 (8)	0.0011 (7)
C2	0.0382 (12)	0.0369 (14)	0.0260 (11)	−0.0032 (8)	0.0175 (9)	−0.0040 (7)
C3	0.0316 (10)	0.0373 (13)	0.0180 (9)	0.0023 (7)	0.0151 (8)	−0.0010 (7)

### Geometric parameters (Å, °)

**Table:**

S1—C1	1.750 (2)	N2—C3	1.359 (3)
S1—S1^i^	2.0713 (14)	N2—H2A	0.86
N1—C2	1.324 (3)	C2—C3	1.329 (3)
N1—C1	1.364 (3)	C2—H2	0.93
N2—C1	1.324 (3)	C3—H3	0.93
			
C1—S1—S1^i^	101.62 (7)	N1—C1—S1	122.51 (16)
C2—N1—C1	102.96 (19)	N1—C2—C3	110.0 (2)
C1—N2—C3	102.12 (19)	N1—C2—H2	125
C1—N2—H2A	128.9	C3—C2—H2	125
C3—N2—H2A	128.9	C2—C3—N2	110.52 (19)
N2—C1—N1	114.4 (2)	C2—C3—H3	124.7
N2—C1—S1	123.13 (18)	N2—C3—H3	124.7
			
C3—N2—C1—N1	0.1 (3)	S1^i^—S1—C1—N1	−93.68 (18)
C3—N2—C1—S1	−179.85 (15)	C1—N1—C2—C3	0.5 (3)
C2—N1—C1—N2	−0.4 (3)	N1—C2—C3—N2	−0.4 (3)
C2—N1—C1—S1	179.60 (17)	C1—N2—C3—C2	0.2 (3)
S1^i^—S1—C1—N2	86.31 (19)		

Symmetry codes: (i) −*x*, *y*, −*z*+1/2.

### Hydrogen-bond geometry (Å, °)

**Table:**

*D*—H···*A*	*D*—H	H···*A*	*D*···*A*	*D*—H···*A*
N2—H2A···S1^ii^	0.86	2.44	3.227 (3)	153

Symmetry codes: (ii) *x*, −*y*, *z*+1/2.
